# Antireflux myoplasty: Endoscopic myoplasty with bilateral sling fiber plication for refractory gastroesophageal reflux disease

**DOI:** 10.1002/deo2.70134

**Published:** 2025-05-07

**Authors:** Hironari Shiwaku, Akio Shiwaku, Hisatoshi Irie, Takayuki Akasaki, Seiya Sato, Nobuhiko Koreeda, Katsudai Shirakabe, Kosuke Yamauchi, Haruhiro Inoue, Suguru Hasegawa

**Affiliations:** ^1^ Department of Gastroenterological Surgery Fukuoka University Faculty of Medicine Fukuoka Japan; ^2^ Digestive Diseases Center Showa University Koto Toyosu Hospital Tokyo Japan

**Keywords:** endoscopy, gastroesophageal junction, gastroesophageal reflux disease, Los Angeles, suture techniques

## Abstract

Endoscopic antireflux therapy is a novel endoscopic treatment for refractory gastroesophageal reflux disease. We developed antireflux myoplasty (AR‐MP), a modified version of antireflux mucoplasty (ARM‐P), in which exposed bilateral sling fibers are sutured directly via endoscopic hand‐suturing. AR‐MP was performed on a 60‐year‐old man, resulting in symptomatic improvement and allowing discontinuation of acid‐suppressive medication 3 months after the procedure. One month postoperatively, endoscopy showed an improvement in the Hill classification from grade 3 to grade 1. Before AR‐MP, endoscopic pressure study integrated system findings showed a maximum intragastric pressure value of 13.7 mmHg, indicating a flat pattern. After AR‐MP, maximum intragastric pressure exceeded 20 mmHg, and the pattern shifted to uphill. AR‐MP is an innovative endoscopic technique that reconstructs the native antireflux mechanism by suturing the sling fibers and reforming the gastroesophageal flap valve. This innovative endoscopic procedure, like ARM‐P, provides immediate symptom relief and represents a breakthrough in the endoscopic treatment of gastroesophageal reflux disease.

## INTRODUCTION

Various endoscopic treatments for refractory gastroesophageal reflux disease (GERD) have been reported by Inoue et al., including antireflux mucosectomy,^1^ antireflux mucosal ablation,^2^ antireflux mucoplasty (ARM‐P),^3^ and ARM‐P with a valve.^4^ Specifically, ARM‐P^3^ involves resecting 1/3‐2/3 of the mucosa at the gastric lesser curvature using endoscopic mucosal resection or endoscopic submucosal dissection (ESD), followed by suturing of the mucosal defect to reconstruct the gastroesophageal junction, leading to immediate treatment effects.

Building upon these approaches, we developed a modified technique, termed antireflux myoplasty (AR‐MP), in which the sling fibers of both the anterior and posterior walls—structures responsible for the physiological prevention of reflux—are exposed and sutured using an endoscopic hand‐suturing (EHS) technique to reconstruct the native antireflux mechanism by reforming the gastroesophageal flap valve. Herein, we report our initial clinical experience with AR‐MP.

## CASE REPORT

### AR‐MP method

**FIGURE 1:**
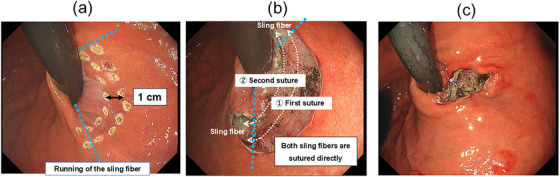
Overview of antireflux myoplasty. (a) The design for mucosal resection is marked using argon plasma coagulation. The short‐axis direction is marked along the lesser curvature so that the inner sides of the anterior and posterior sling fibers are exposed. The long‐axis direction is marked approximately 1 cm from the gastroesophageal junction as a reference. (b) The mucosa of the lesser curvature is resected using endoscopic submucosal dissection, exposing bilateral sling fibers. Endoscopic hand‐suturing is performed to approximate the sling fibers, completing the myoplasty. (c) Endoscopic finding after myoplasty. Mucosal closure after sling fiber suturing is optional. *Note: The images shown in this figure are from a different case than the one described in the manuscript, as key explanatory images were not available from the present case*.

The AR‐MP method is shown in Figure 1 and the Video S1. The procedure was performed under general anesthesia with carbon dioxide (CO₂) insufflation. An endoscope (GIF‐H290T; Olympus) with a transparent hood attachment (D‐201‐11804; Olympus) and an overtube were used. The gastric cardia was observed retrograde to confirm the running of the sling fibers. The mucosal resection area was marked using argon plasma coagulation, ensuring exposure of the inner part of the sling fibers on both the anterior and posterior walls. ESD was then used to resect the mucosa, exposing both sling fibers. The dissection depth was just above the muscle layer to ensure a clear view of the muscle fibers. Hemostasis was performed if needed for penetrating vessels adjacent to the sling fibers. Myoplasty was performed using the endoscopic ligation technique (ELT; Figure S1)^5^ with an endoscopic needle holder (FG‐260Q; Olympus) and 4‐0 absorbable sutures (MONO STINGER; Nitcho Kogyo Co., Ltd.; 180 cm in length). Suturing proceeded from anal to oral directions to approximate the sling fibers, ensuring the formation of an effective gastroesophageal flap valve and closure of the gastroesophageal junction. Mucosal closure was optional.

### Patient

A 60‐year‐old man experienced acid reflux for 1 year. Endoscopic examination diagnosed Los Angeles classification grade C reflux esophagitis. Although continuous oral administration of 20 mg vonoprazan or 20 mg esomeprazole for more than eight weeks, along with multiple other oral medications for GERD, improved the patient's erosive esophagitis, his symptoms persisted. Therefore, he sought further examination and treatment at our hospital. At that time, even under acid‐suppressive therapy (esomeprazole 20 mg), his F‐scale score was 9 points (acid reflux symptoms: 2 points). Endoscopic evaluation revealed Los Angeles classification grade N reflux esophagitis. The gastroesophageal junction was observed as cardiac opening (CO)‐3, sliding hernia (SH)‐1 in accordance with Inoue et al.’s assessment of the gastroesophageal junction,^6^ and Hill classification grade 3. Even with insufflation, CO₂ escaped from the stomach into the esophagus, preventing sufficient gastric distension.

**FIGURE 2:**
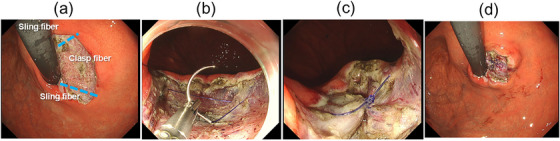
Antireflux myoplasty (AR‐MP) in the present case. (a) Following mucosal resection, bilateral sling fibers are visible. (b) Suture placement on both the anterior and posterior sling fibers. (c) In this case, myoplasty was performed using the endoscopic ligation technique to tightly approximate the bilateral sling fibers. (d) Endoscopic finding after myoplasty. Mucosal suturing was not performed after completing AR‐MP in this case.

**FIGURE 3:**
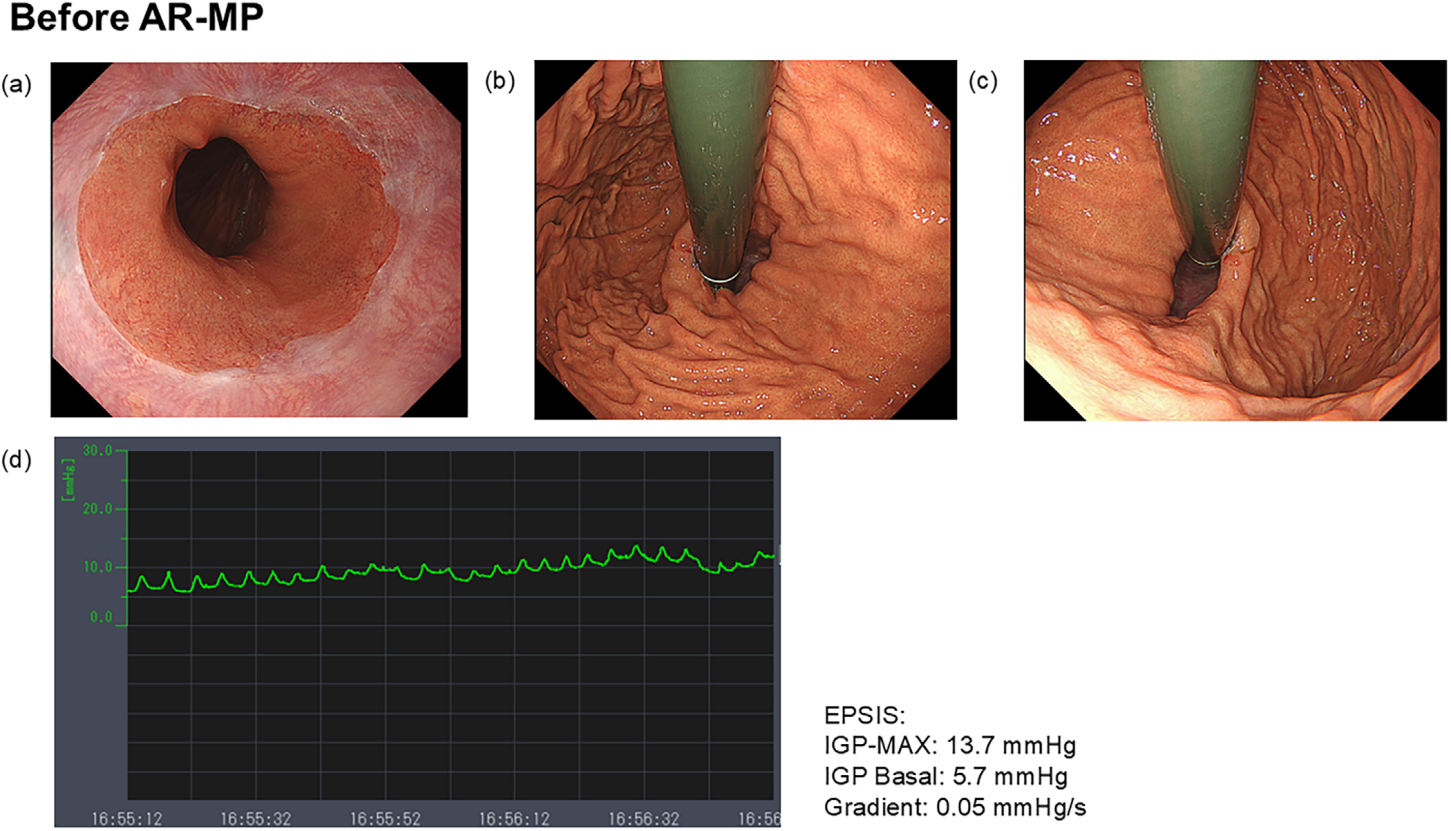
Endoscopic and endoscopic pressure study integrated system (EPSIS) findings before antireflux myoplasty. Endoscopic findings (a–c): During insufflation, CO₂ escaped from the stomach into the esophagus, preventing adequate gastric distension. Additionally, the gastroesophageal junction remained widely open. EPSIS findings (d): maximum intragastric pressure was 13.7 mmHg, and the pressure gradient was 0.05, indicating a flat pattern.

Endoscopic pressure study integrated system^7^, ^8^ findings showed a maximum intragastric pressure (IGP‐MAX) of 13.7 mmHg; basal: 5.7 mmHg, gradient: 0.05 mmHg/s, indicating a flat pattern. Computed tomography confirmed the absence of a hiatal hernia. Under acid‐suppressive therapy (esomeprazole 20 mg), 24‐h impedance‐pH testing indicated an acid exposure time percentage of 2.0%, a non‐acid exposure time percentage of 4.5%, and a DeMeester score of 13.9. High‐resolution esophageal manometry did not indicate esophageal motility disorder. On the basis of these findings, a diagnosis of refractory GERD was confirmed, and AR‐MP was performed using the aforementioned method (Figures 2 and 3). The total procedure time was 150 min, consisting of 70 min for removal of the mucosa and submucosa to expose bilateral sling fibers using the ESD technique, 55 min for placing two stitches using ELT, and 25 min for an unsuccessful attempt to place one stitch.

**FIGURE 4:**
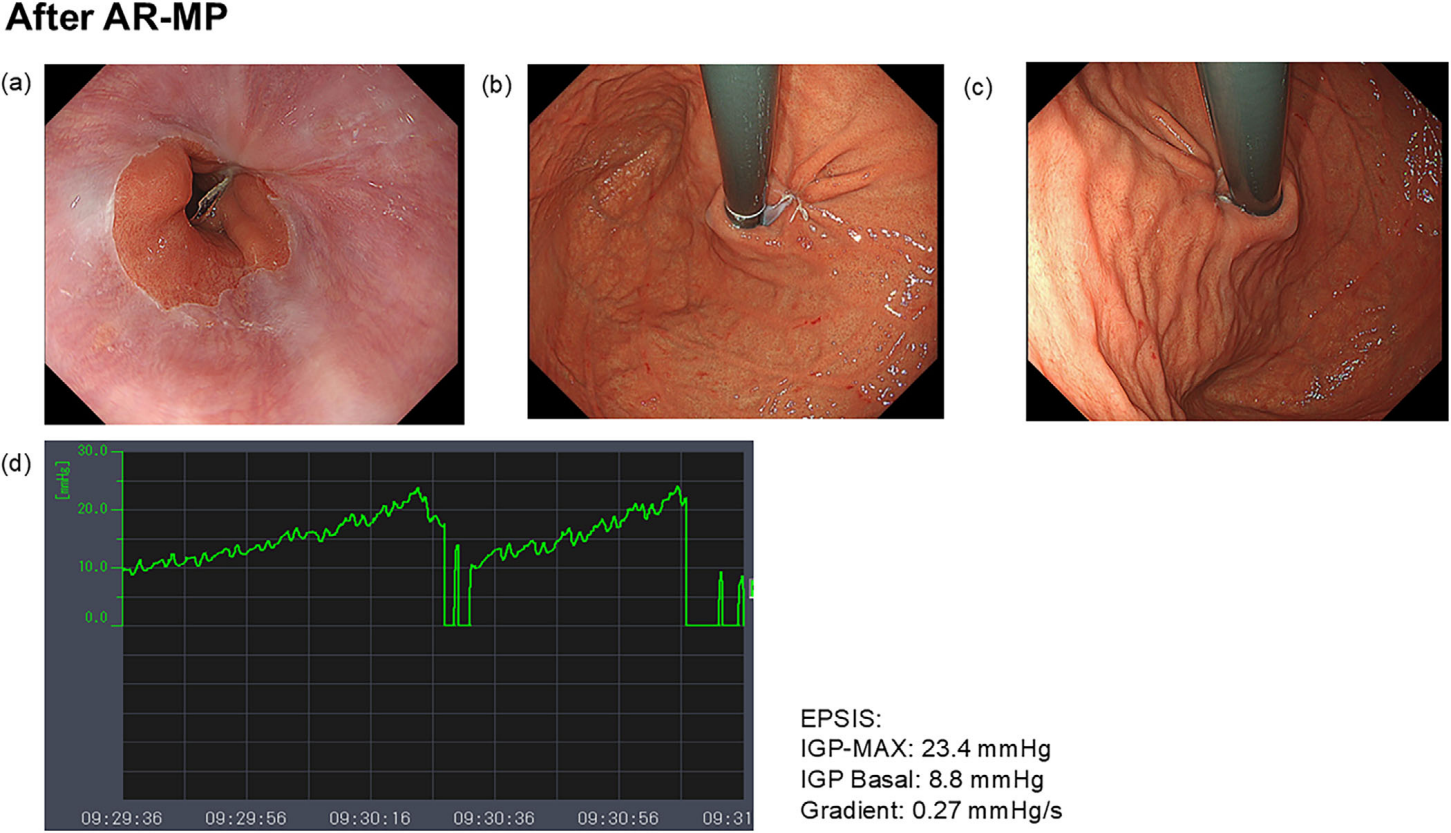
Endoscopic and EPSIS findings after AR‐MP. Endoscopic findings (a–c): The bilateral sling fibers were tightly approximated, forming a well‐defined gastroesophageal flap valve, thereby reinforcing Phase I of the antireflux mechanism. Furthermore, gastric distension with CO₂, which was previously not possible before AR‐MP, was successfully achieved after AR‐MP. EPSIS findings (d): The IGP‐MAX exceeded 20 mmHg, indicating a transition to an uphill pattern. These findings were consistent with the endoscopic observations shown in Figure 4. AR‐MP, antireflux myoplasty; CO₂, carbon dioxide; EPSIS, endoscopic pressure study integrated system; IGP‐MAX, maximum intragastric pressure.

One month postoperatively, the patient continued taking vonoprazan 20 mg. His symptoms had gradually improved, and he was satisfied with the treatment outcome. His F‐scale score had decreased to 6 points (acid reflux symptoms: 0 points). Endoscopic findings showed no signs of reflux esophagitis (Los Angeles classification grade N) and improvement of the gastroesophageal junction to CO‐0, SH‐0. The Hill classification had also improved to grade 1. Endoscopic Pressure Study Integrated System (EPSIS) findings showed an uphill pattern, with an IGP‐MAX of 23.4 mmHg, a basal pressure of 8.8 mmHg, and a gradient of 0.27 mmHg/s (Figure 4). Based on the improvement in symptoms and endoscopic findings, vonoprazan was definitively discontinued at the 1‐month follow‐up.

At the 3‐month follow‐up, the patient's symptoms were well controlled without the use of acid‐suppressive therapy. His F‐scale score had decreased to 4 (acid reflux symptoms: 0).

## DISCUSSION

### Sling fibers and clasp fibers as key components of the antireflux mechanism

Sling fibers and clasp fibers are essential components of the gastroesophageal antireflux mechanism. Inoue et al. proposed the “phase concept” regarding the antireflux mechanism.^9^ According to this concept, the antireflux mechanism of the esophagus and stomach consists of three phases: Phase I (gastric phase), Phase II (lower esophageal sphincter phase), and Phase III (esophageal clearance phase). Sling fibers and clasp fibers are included in Phase I. Generally, endoscopic antireflux procedures aim to restore Phase I.

Inoue et al. have reported the following innovative endoscopic treatments as methods for restoring Phase I of the antireflux mechanism: antireflux mucosectomy,^1^ antireflux mucosal ablation,^2^ ARM‐P,^3^ and ARM‐P with a valve.^4^ AR‐MP is a modified version of ARM‐P. AR‐MP reconstructs Phase I by directly suturing (myoplasty) the bilaterally important sling fibers of the antireflux mechanism, aiming to achieve endoscopic fundoplication. Because approximation of the sling fibers can be observed directly during the procedure, a high therapeutic effect can be expected. Indeed, in the present case, improvement in GERD symptoms and discontinuation of acid‐suppressive medication were confirmed, along with objective improvement of Phase 1 findings evaluated by EPSIS.

### Technical challenges and future prospects of AR‐MP

One of the main challenges of AR‐MP is its technical difficulty. Compared with other gastrointestinal structures such as the colon, the gastric muscle layer is more robust, and its mucosal distensibility is limited, making wound closure after ESD or endoscopic mucosal resection more challenging. In ARM‐P, a dead space‐eliminating technique^10^ has been used to achieve suture closure of large defects resulting from resection of one‐third to two‐thirds of the mucosa at the gastric lesser curvature. However, AR‐MP requires exposing the bilateral sling fibers, which necessitates the closure of a wider wound than that with ARM‐P. Suturing such a large defect involving muscular layers is technically challenging with conventional clips.

In AR‐MP, this issue is addressed using EHS. In the present case, suturing with ELT^5^ enabled endoscopic fundoplication. Theoretically, continuous suturing with EHS can also be applied to AR‐MP, and future clinical reports on alternative methods to ELT are anticipated.

In addition, mucosal closure was not performed in this case. Suturing the muscle layer approximates the mucosa and achieves a near‐closure state, and additional mucosal closure was considered unnecessary as it would have required extra time and cost without providing significant additional benefit to the patient. Furthermore, performing mucosal closure could have confounded the evaluation by mixing the effects of myoplasty and mucosal closure, making it difficult to accurately assess the true efficacy of myoplasty. Therefore, at this stage, the procedure has been standardized without mucosal closure.

Another challenge of AR‐MP is that it requires more procedural time than ARM‐P. With currently available endoscopic tools, suturing with EHS takes longer than closure with clips. However, if advances in techniques and instruments overcome this limitation, AR‐MP could become more widely adopted.

In conclusion, AR‐MP is a groundbreaking technique that enables direct visualization of the bilateral sling fibers responsible for the antireflux mechanism, reduces the gap between them, and reforms the gastroesophageal flap valve, thereby allowing anatomical restoration of the native antireflux mechanism. This procedure is expected to provide high therapeutic efficacy immediately after treatment, similar to ARM‐P, in patients with refractory GERD. Further accumulation of clinical cases is needed in the future.

## CONFLICT OF INTEREST STATEMENT

Outside of this research, Hironari Shiwaku has served as an advisor for Olympus. Haruhiro Inoue has served as an advisor for Olympus and Top and has received educational grants from Olympus and Takeda. The other authors declare no conflict of interest.

## ETHICS STATEMENT

The treatment was performed in accordance with the principles of the Declaration of Helsinki.

## PATIENT CONSENT STATEMENT

Written informed consent was obtained from the patient.

## Supporting information



FIGURE S1 Procedure for performing myoplasty using the endoscopic ligation technique.
(a) Both ends of the suture are extended outside the body.
(b) An extracorporeal knot is created.
(c) The suture is grasped securely with a needle holder inserted through the forceps opening, and any excess suture is trimmed. While the surgeon's left hand pulls the suture, the right hand inserts the endoscope, guiding the knot through the overtube to its target location. For further details, please refer to Shiwaku et al., DEN Open, 2024.

VIDEO S1 The video shows the novel AR‐MP technique described in this report. AR‐MP, antireflux myoplasty.
