# Supplementary figures and images for: Antireflux myoplasty: Endoscopic myoplasty with bilateral sling fiber plication for refractory gastroesophageal reflux disease
Source: DEN Open. 2025 May 7;6(1):e70134. doi: 10.1002/deo2.70134 (PMC12056592; doi:10.1002/deo2.70134)

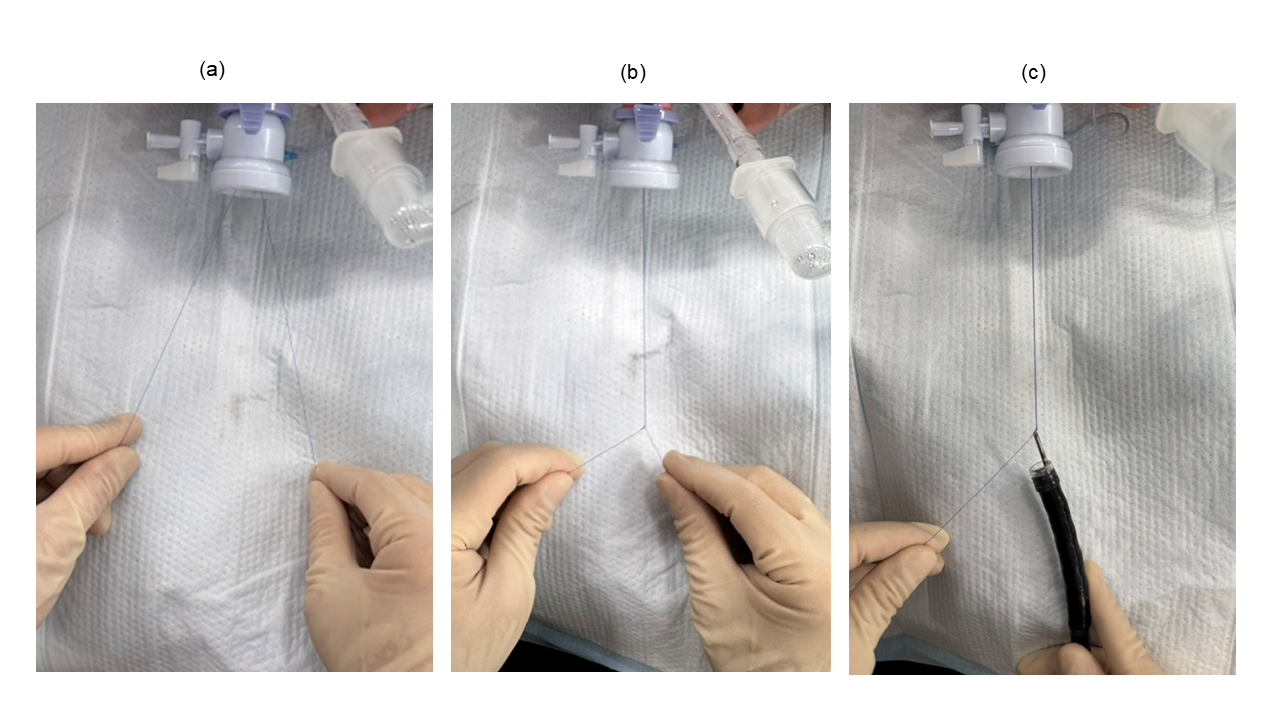

Supplement: Supplementary file 1 — FIGURE S1 Procedure for performing myoplasty using the endoscopic ligation technique. (a) Both ends of the suture are extended outside the body. (b) An extracorporeal knot is created. (c) The suture is grasped securely with a needle holder inserted through the forceps opening, and any excess suture is trimmed. While the surgeon's left hand pulls the suture, the right hand inserts the endoscope, guiding the knot through the overtube to its target location. For further details, please refer to Shiwaku et al., DEN Open, 2024. [file DEO2-6-e70134-s002.TIF]
